# Circ‐UBAP2 functions as sponges of miR‐1205 and miR‐382 to promote glioma progression by modulating STC1 expression

**DOI:** 10.1002/cam4.3759

**Published:** 2021-02-05

**Authors:** Jianxin Wang, Tianxiao Li, Bin Wang

**Affiliations:** ^1^ Department of Neurosurgery Henan Provincial People’s Hospital (Zhengzhou University People’s Hospital) Zhengzhou Henan China; ^2^ Department of Neurosurgery Henan Provincial Cerebrovascular Hospital Zhengzhou Henan China; ^3^ Department of Neurosurgery Henan University People’s Hospital Kaifeng Henan China; ^4^ Department of Cerebrovascular Henan Provincial Cerebrovascular Hospital Zhengzhou Henan China; ^5^ Department of Cerebrovascular Henan Provincial Cerebrovascular Hospital Kaifeng Henan China

**Keywords:** circ‐UBAP2, Glioma, GPRC5A, miR‐1205, miR‐382

## Abstract

**Background:**

Circular RNAs (circRNAs) exert vital functions in glioma pathogenesis. CircRNA ubiquitin‐associated protein 2 (circ‐UBAP2, hsa_circ_0008344) has been illuminated as a tumor driver in glioma. Nevertheless, the mechanisms underlying the oncogenic regulation of circ‐UBAP2 in glioma are still undefined.

**Methods:**

Circ‐UBAP2, miR‐1205, miR‐382, and GPRC5A were quantified using qRT‐PCR and western blot. Cell viability was detected using a CCK‐8 assay. Cell migration and invasion were measured using the would‐healing and transwell assays. Flow cytometry and colony formation assay were applied to evaluate cell apoptosis and colony formation, respectively. The xenograft model assays were used to examine the impact of circ‐UBAP2 on tumorigenic effect in vivo. Direct relationships among circ‐UBAP2, miR‐1205, miR‐382, and GPRC5A were confirmed using dual‐luciferase reporter assays.

**Results:**

Circ‐UBAP2 expression was upregulated in glioma. The reduced level of circ‐UBAP2 hampered cell proliferation, migration, invasion, and enhanced apoptosis in vitro and weakened tumor growth in vivo. Mechanistically, circ‐UBAP2 directly bound to miR‐1205 and miR‐382. miR‐1205 and miR‐382 mediated the regulation of circ‐UBAP2 silencing on glioma cell behaviors. Moreover, GPRC5A was a functional target of miR‐1205 and miR‐382 in regulating glioma cell behaviors. Furthermore, circ‐UBAP2 mediated GPRC5A expression through miR‐1205 or miR‐382 in glioma cells.

**Conclusion:**

Our current findings identified that circ‐UBAP2 silencing impeded glioma malignant progression partially by downregulating GPRC5A through targeting miR‐1205 and miR‐382.

## Graphical abstract

STC1 was a direct target of miR‐1205 and miR‐382 in glioma cells.

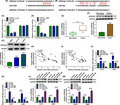

## 1 INTRODUCTION

Glioma remains the most prevalent primary brain malignancy globally.^1^, ^2^ Despite advances in therapeutic approaches, effective treatments against glioma are still limited.^3^ A further understanding of what drives the pathology of glioma would provide a novel opportunity to develop better therapeutic interventions.

Covalently closed circular RNAs (circRNAs) have vital noncoding functions in cancer biology.^4^ Some circRNAs have been demonstrated as posttranscriptional regulators of gene expression by sequestering microRNAs (miRNAs) during glioma development process.^5^ Li et al. discovered that hsa_circ_0046701 operated as a contributor in glioma carcinogenesis by controlling ITGB8 expression through miR‐142‐3p.^6^ Duan et al. identified that hsa_circ_0074362 accelerated the development of aggressive glioma by reducing the suppression of miR‐1236‐3p on homeobox B7 expression.^7^ As for circRNA ubiquitin‐associated protein 2 (circ‐UBAP2, hsa_circ_0008344), an overexpressed circRNA in glioma, it was identified as a strong tumor driver in glioma.^8^ Nevertheless, the mechanisms underlying the oncogenic role of circ‐UBAP2 in glioma largely remain to be elucidated.

miRNAs are the best‐known noncoding RNAs that have been implicated in human cancers, including glioma.^9^, ^10^ miR‐1205 and miR‐382 were reported to be involved in the development of glioma.^11^, ^12^, ^13^, ^14^ Moreover, these reports demonstrated miR‐1205 or miR‐382 as a target of circRNAs in regulating glioma progression. However, it remains unknown whether the oncogenic effect of circ‐UBAP2 on glioma is mediated by miR‐1205 or miR‐382.

Here, our data supported the oncogenic activity of circ‐UBAP2 in glioma in vitro and in vivo. Consequently, we explored the mechanisms underlying the oncogenic role of circ‐UBAP2, with the hope that such work might provide new circRNA/miRNA/mRNA regulatory networks in glioma development.

## 2 MATERIALS AND METHODS

### 2.1 Specimen collection and cell culture

The current study included an analysis of 31 tumor tissues and 31 healthy nervous tissues that were obtained from patients with glioma and healthy volunteers, respectively, at Henan Provincial People’s Hospital. Detailed questionnaires, including informed consent, were given by these participators. This study was approved by the Ethics Committee of Henan Provincial People’s Hospital.

LN18 and A172 cell lines (National Center for Cell Science, Pune, India) and U251 glioma cells (Bnbio) were maintained at log‐growth in DMEM (Gibco) plus 10% FBS (Bovogen Biologicals) at 37°C with 5% CO_2_. Available normal human astrocytes (NHA, Cell Systems) were used as a control in this study and propagated using complete Astrocyte Basal Medium provided by Cell Systems.

### 2.2 Lentiviral transduction and transient transfection of cells

shRNA‐circ‐UBAP2 lentiviruses (sh‐circ‐UBAP2 and sh‐circ‐UBAP2#1) or control vector lentiviruses (sh‐NC) were used to transduce U251 cells as recommended by the manufacturing (Hanbio). Stable U251 cell lines were obtained by the selection of puromycin (Yesen).

siRNAs against circ‐UBAP2 (si‐circ‐UBAP2, 5'‐CAGACACUAGACAUGAGCCUU‐3' and si‐circ‐UBAP2#1, 5'‐GCAGACACUAGACAUGAGCCU‐3') or nontarget siRNA (si‐NC, 5'‐GGUACACCCUCCAUGGUAAUU‐3'), miR‐1205 mimic (5'‐GAGUUUCGUUUGGGACGUCU‐3'), miR‐382 mimic (5'‐GCUUAGGUGGUGCUUGUUGAAG‐3') or the scrambled oligonucleotide negative control (miR‐NC mimic, 5'‐CGAUCGCAUCAGCAUCGAUUGC‐3'), miRNA inhibitors (anti‐miR‐1205, 5'‐AGACGUCCCAAACGAAACUC‐3', and anti‐miR‐382, 5'‐CUUCAACAAGCACCACCUAAGC‐3') or negative control inhibitor (anti‐miR‐NC, 5'‐CUAACGCAUGCACAGUCGUACG‐3') were synthesized by Hanbio. Circ‐UBAP2 overexpressing plasmid (circ‐UBAP2) was generated by cloning the sequence of circ‐UBAP2 into the pCD5‐ciR vector (Geneseed, Guangzhou, China) with EcoR I and BamH I restriction sites, with a nontarget vector (Vector) as the control. The sequence of GPRC5A (Accession: NM_003979.4) was inserted into the pcDNA3.1 vector (Promega) with BamH I and Xho I sites to produce the recombinant GPRC5A overexpressing plasmid (GPRC5A), and negative plasmid (pcDNA) was used as the control. Transient transfections were done with the indicated plasmid (100 ng) or oligonucleotide (50 nM) using Lipofectamine 3000 (Invitrogen). Transfected cells were harvested 48 h later for further exploration.

### 2.3 RNA extraction and Ribonuclease R (RNase R) assay

RNA extraction from tissues and cells was done using the miRNeasy Mini Kit as per the manufacturing protocols (Qiagen). RNA with a purity of 1.96–2.04 (A260/280) was used for further experiments.

RNase R treatment was carried out by adding 10 U RNase R (Geneseed) into a 20 µl reaction containing 3 µg of total RNA, followed by RNA purification with phenol–chloroform extraction after 20 min incubation at 37°C.

### 2.4 qRT‐PCR

cDNA was generated using 10 ng of total RNA with the IScript cDNA Kit (Bio‐Rad), which was then used to quantify circ‐UBAP2 and mRNAs using qRT‐PCR using iTaq SYBR Green (Bio‐Rad) on the Chromo4 PCR System (Bio‐Rad). The expression of miR‐1205 and miR‐382 was quantified using the miScript RT Kit and miScript SYBR Green as per the manufacturing guidance (Qiagen). Results were determined using the cycle to threshold (2^‒ΔΔCt^) method,^15^ normalizing to β‐actin or U6 housekeeping gene. The primers were synthesized using Hanbio and their details were shown in Table 1.

**TABLE 1:** Primers sequences used for qRT‐PCR amplification

Primers for qRT‐PCR (5'‐3')		
Circ‐UBAP2	Forward	AGAGTCAGCTCCAGGAACCA
	Reverse	GCAGGAGGTAATGACGGAAG
UBAP2 linear mRNA	Forward	CCTGCAGTCTGACAAGCTCA
	Reverse	TGGTTCCTGGAGCTGACTCT
GRPC5A	Forward	TACGGGAACAGTTTGCCTCC
	Reverse	GAGTTGCCTGAGACTCCCAC
miR‐1205	Forward	GCCGAGCGTTTGGGACGTCT
	Reverse	CAGTGCGTGTCGTGGAGT
miR‐382	Forward	GCCGAGGCTTAGGUGGTGC
	Reverse	CAGTGCGTGTCGTGGAGT
U6	Forward	CTCGCTTCGGCAGCACA
	Reverse	AACGCTTCACGAATTTGCGT
β‐actin	Forward	CTCGCCTTTGCCGATCC
	Reverse	GGGGTACTTCAGGGTGAGGA
GAPDH	Forward	GAATGGGCAGCCGTTAGGAA
	Reverse	AAGCATCACCCGGAGGAG

### 2.5 Subcellular fractionation

The assays were done based on the standard methods.^16^ A cytoplasmic and nuclear RNA purification kit was applied to extract cytoplasmic and nuclear RNA, following the manufacturing protocols (Norgen Biotek). GAPDH and U6 were used as the cytoplasmic and nuclear controls, respectively.

### 2.6 Cell viability assay

Transfected cells were seeded at 5000 cells/well in 96‐well plates and then incubated with Cell Counting Kit‐8 (CCK‐8, 10 µl per well) solution as recommended by the manufacturers (Genomeditech). Data were obtained by gauging absorbance using a Viktor X3 reader (Perkin Elmer) at 450 nm. The untransfected cells were defined as the control group, and cell viability was presented as percentage of control cells.

### 2.7 Colony formation assay

About 150 transfected cells were plated in per well of six‐well plates and maintained at 37°C for 2 weeks. Following the staining with 0.5% crystal violet (Solarbio), the number of colonies (>50 cells) were manually counted.

### 2.8 Cell apoptosis assay

Propidium iodide (PI) and FITC‐labeled Annexin V (Invitrogen) were applied to evaluate the cells that were undergoing apoptosis.^17^ About 1.0 × 10^5^ transfected cells were stained with 5 µl of Annexin V‐FITC and 10 µl of PI. The apoptotic cells were analyzed using a flow cytometer as reported.^18^

### 2.9 Transwell assay

24‐Transwell inserts (8 µm pores, Corning) were used to assess cell migration, and transwell inserts coated with Matrigel (Corning) were applied for cell invasion measurement. Transfected cells were plated onto the transwell inserts at 5.0 × 10^4^ for migration assays and 1.0 × 10^5^ for invasion assays. Twenty‐four hours later, the cells on the undersurface of the insert membranes were photographed and counted in five random fields under a 100× magnification microscope (Nikon).

### 2.10 Wound‐healing assay

A total of 1.0 × 10^6^ transfected cells were seeded in per well of six‐well plates. When the cells grew to 95% confluence, a scratch was made using a sterile pipette tip on the cell monolayer. After 24 h of culture, migration distance was observed under a 40× magnification microscope and analyzed using Image J software (Rawak).

### 2.11 Dual‐luciferase reporter assay

Analysis of the targeted miRNAs of circ‐UBAP2 was carried out using the CircInteractome online database. miRNA‐binding sites were predicted using the starBase software. The sequence of circ‐UBAP2 and its mutation in the two seed sites were individually cloned into the pmirGLO vector (Promega). The fragments of GPRC5A 3'UTR harboring the two putative target sequences for miRNAs or miss‐matched miRNAs‐binding sites were cloned into the pmirGLO vector, respectively. Each reporter construct (100 ng) was individually transfected into A172 and U251 cells with the mimic of miR‐1205, miR‐382 or negative control at 50 nM using Lipofectamine 3000. Luciferase activities were gauged by the Promega Dual‐luciferase Assay System.

### 2.12 Western blot for GPRC5A level

Proteins (100 µg per lane) isolated from the cells with the RIPA lysis buffer (Sbjbio) were separated by electrophoresis using Mini‐Protean TGX Precast gels (Bio‐Rad). After being electroblotted onto the nitrocellulose membranes (Bio‐Rad), the blots were probed with anti‐CyclinD1 (MA5‐16356, Invitrogen), anti‐MMP9 (MA5‐32705, Invitrogen), anti‐GPRC5A (A304‐441A, Invitrogen) and anti‐GAPDH (PA1‐16777, Invitrogen), followed by the incubation with secondary IgG antibody coupled by horseradish peroxidase (G‐21234, Invitrogen). The blots were developed using the Immun‐Star HRP Lumino‐Enhancer (Bio‐Rad) and band intensity was evaluated with the Image J software.

### 2.13 Animal studies

Animal experiments were done based on an approved protocol by the Ethics Committee of Animal Care and Use of Henan Provincial People’s Hospital. The xenograft model was generated by subcutaneous implantation of 1.0 × 10^7^ sh‐circ‐UBAP2‐, sh‐circ‐UBAP2#1‐transduced or sh‐NC‐infected U251 cells into the left flank of female BALB/c mice aged with 6‐week‐old (*n* = 6 per group, ALF Biotechnology, Nanjing, China). Additionally, intratumoral injection with circ‐UBAP2 overexpressing plasmid, negative control Vector or PBS was performed every 3 days after 5 days of cell implantation. Tumor volume was periodically estimated by using the formula length × width^2^ × 0.5. Twenty‐six days later, the xenograft tumors were harvested from the experimental mice.

### 2.14 Statistical analysis

Data were shown as the mean ± SD of separate experiments (*n* ≥ 3). The Spearman’s test was used to evaluate the correlations among circ‐UBAP2, miR‐1205, miR‐382, GPRC5A expression levels in tumor specimens. Statistical significance was evaluated using a Student’s *t*‐test, Mann–Whitney *U* test, or ANOVA assuming variance with *p* < 0.05 considered significant.

## 3 RESULTS

### 3.1 Circ‐UBAP2 expression was upregulated in glioma tissues and cells

**FIGURE 1:**
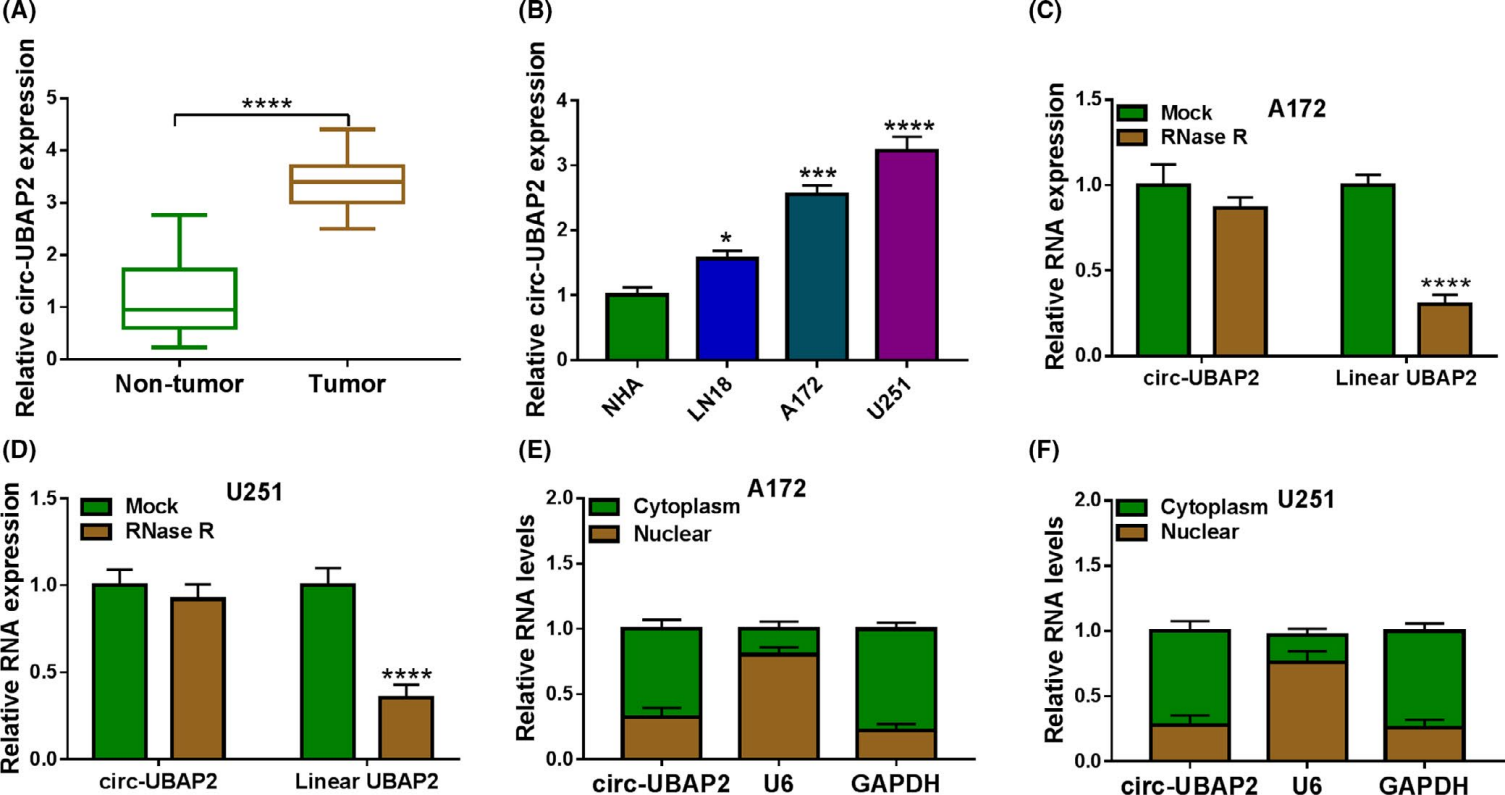
Circ‐UBAP2 was overexpressed in glioma tissues and cells. Relative circ‐UBAP2 level by qRT‐PCR in 31 tumor tissues and 31 healthy tissues (A), NHA, LN18, A172, and U251 cells (B). (C, D) The levels of circ‐UBAP2 and corresponding linear transcript (Linear UBAP2) in total cellular RNA incubated with RNase R or Mock. (E, F) Subcellular location assays in A172 and U251 cells. *p < 0.05, ***p < 0.001 or ****p < 0.0001

In contrast to the corresponding negative control, circ‐UBAP2 level was dramatically elevated in glioma tissues and cell lines (Figure 1A,B). RNase R assays showed that the incubation of RNase R led to a clear reduction in the level of the corresponding linear transcript (Linear UBAP2), and circ‐UBAP2 was resistant to RNase R, demonstrating the stability of circ‐UBAP2 (Figure 1C,D). Additionally, subcellular localization analysis showed that circ‐UBAP2 was mainly localized in the cytoplasm of A172 and U251 cells (Figure 1E,F). These data together indicated that circ‐UBAP2 was overexpressed in glioma tissues and cells.

### 3.2 Silencing of circ‐UBAP2 hindered cell proliferation, migration, invasion, and enhanced apoptosis in vitro

**FIGURE 2:**
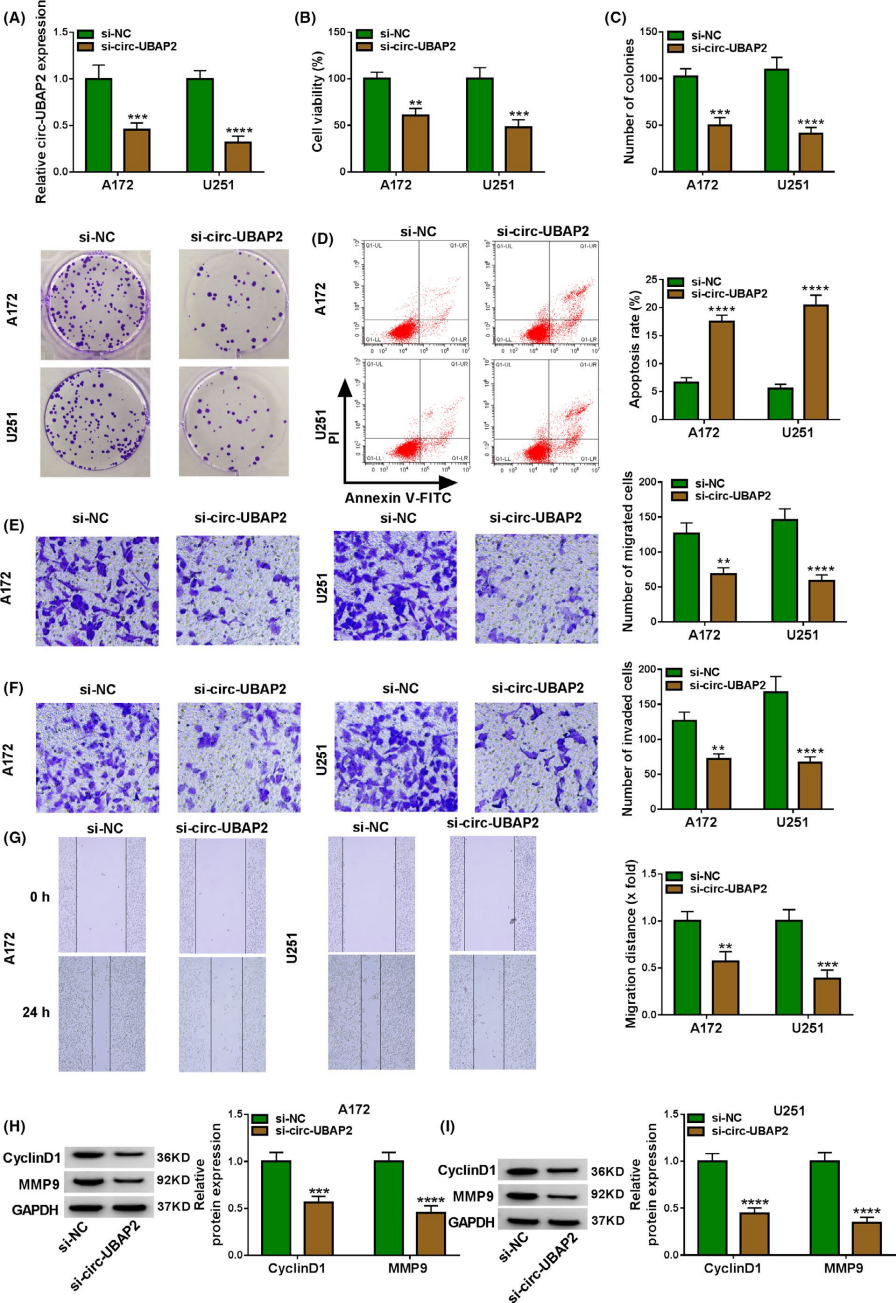
Circ‐UBAP2 silencing repressed cell proliferation, migration, and invasion, and enhanced apoptosis in vitro. A172 and U251 cells were transfected with si‐NC or si‐circ‐UBAP2. (A) Circ‐UBAP2 level was assessed by qRT‐PCR. (B) Cell viability was gauged using a CCK‐8 assay. (C) Colony formation was evaluated using colony formation assay. (D) Cell apoptosis was detected using flow cytometry. (E, F) Cell migration and invasion were gauged using transwell assay. (G) Cell migration was monitored using wound‐healing assay. (H, I) The levels of CyclinD1 and MMP9 by western blot. **p < 0.01, ***p < 0.001, or ****p < 0.0001

To test the functional role of circ‐UBAP2 on glioma progression, we performed “phenocopy” silencing using siRNAs targeting circ‐UBAP2 (si‐circ‐UBAP2 and si‐circ‐UBAP2#1). In contrast, si‐circ‐UBAP2 or si‐circ‐UBAP2#1 triggered a striking downregulation in the level of circ‐UBAP2 in both A172 and U251 cell lines (Figure 2A and Figure [Link], [Link], [Link], [Link], [Link], [Link], [Link], [Link]A). Functional analyses showed that the reduced circ‐UBAP2 expression repressed cell viability (Figure 2B and Figure S1B), colony formation (Figure 2C and Figure S1C), and enhanced cell apoptosis (Figure 2D and Figure S1D), as well as hindered cell migration and invasion (Figure 2E,G, and Figure S1E,G). Moreover, the downregulation of circ‐UBAP2 resulted in decreased levels of CyclinD1 and MMP9 in the two cell lines (Figure 2H,I). To validate that the alteration of cell functional behaviors was veritably induced by si‐circ‐UBAP2, we then carried out a rescue experiment by upregulating circ‐UBAP2 with circ‐UBAP2 overexpressing plasmid in si‐circ‐UBAP2‐transfected cells (Figure [Link], [Link], [Link], [Link], [Link], [Link], [Link], [Link]A). As expected, the restored expression of circ‐UBAP2 dramatically counteracted the regulatory impact of si‐circ‐UBAP2 on cell behaviors in both cell lines (Figure S2B–G). Together, these results suggested that circ‐UBAP2 silencing regulated the functional behaviors of glioma cells in vitro.

### 3.3 Circ‐UBAP2 directly targeted miR‐1205 and miR‐382

**FIGURE 3:**
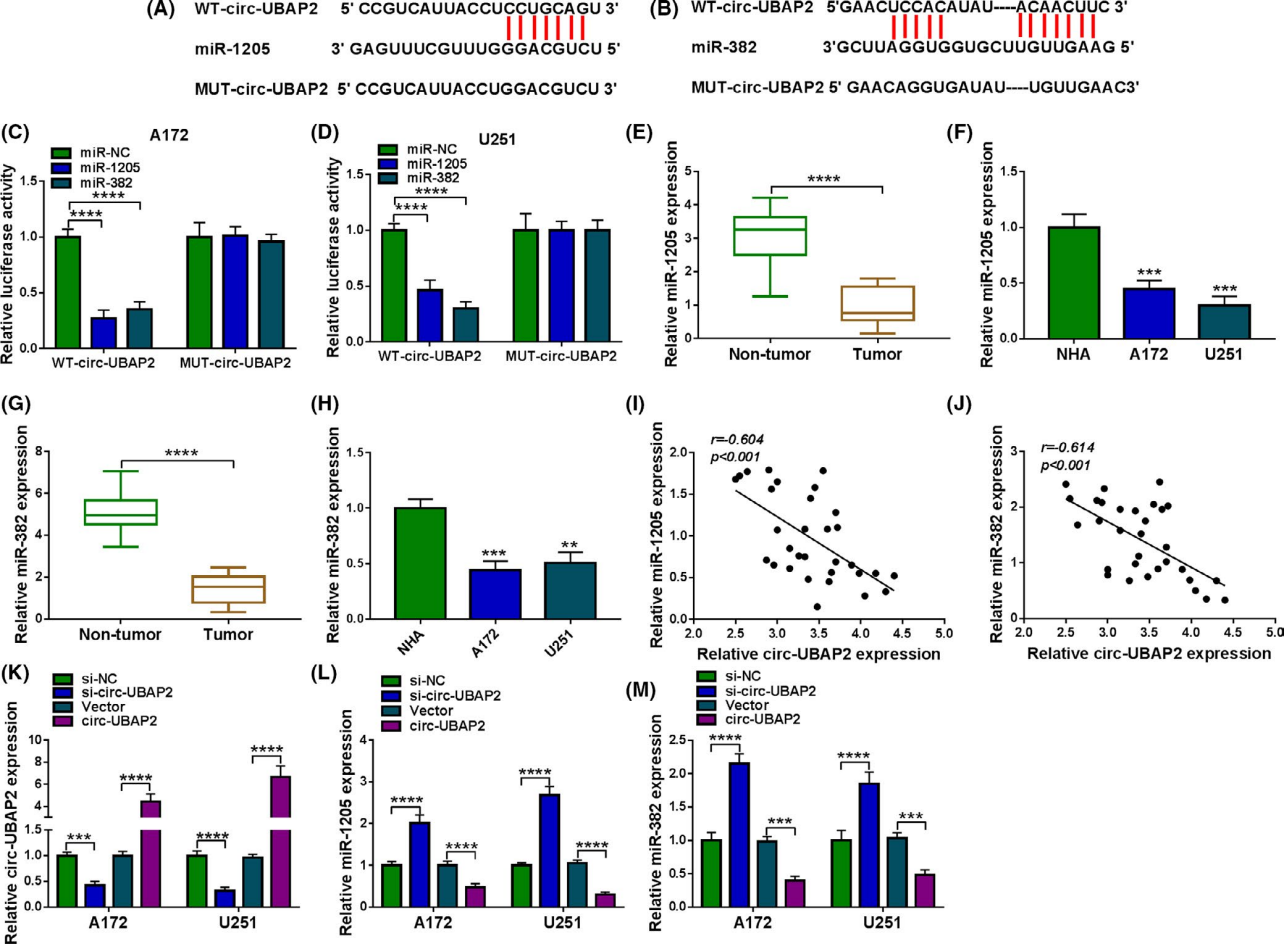
Circ‐UBAP2 directly bound to miR‐1205 and miR‐382 in both A172 and U251 cells. (A, B) Schematic of the putative target regions for miR‐1205 and miR‐382 within circ‐UBAP2 and the mutations of the seed region. (C, D) Dual‐luciferase reporter assays in both A172 and U251 cells. The levels of miR‐1205 (E, F) and miR‐382 (G, H) by qRT‐PCR in 31 tumor tissues and 31 corresponding healthy tissues, and NHA, LN18, A172, and U251 cells. (I, J) Correlation between circ‐UBAP2 expression and miR‐1205 or miR‐382 level in glioma tissues using the Spearman’s test. The expression levels of circ‐UBAP2 (K), miR‐1205 (L) and miR‐382 (M) in A172 and U251 cells transfected with si‐NC, si‐circ‐UBAP2, Vector or circ‐UBAP2 (circ‐UBAP2 overexpressing plasmid). **p < 0.01, ***p < 0.001, or ****p < 0.0001

To elucidate the mechanism of circ‐UBAP2 function on glioma progression, we undertook to search its targeted miRNAs using the prediction tool CircInteractome. Among these candidates, we selected seven miRNAs that were related to glioma pathogenesis. Our data showed that the levels of miR‐1205 and miR‐382 were the most significantly downregulated in the circ‐UBAP2‐overexpressing U251 cells (Figure [Link], [Link], [Link], [Link], [Link], [Link], [Link], [Link]). We thus selected miR‐1205 and miR‐382 for further analysis. The predicted data showed that circ‐UBAP2 contained two putative target sequences for miR‐1205 and miR‐382, respectively (Figure 3A,B). To ascertain this, we carried out dual‐luciferase assays using circ‐UBAP2 luciferase reporter (WT‐circ‐UBAP2) and the mutation of the seed regions (MUT‐circ‐UBAP2). The transfection of WT‐circ‐UBAP2 in the presence of miR‐1205 or miR‐382 mimic triggered a striking reduction of relative luciferase activity, and the effects were dramatically abrogated by MUT‐circ‐UBAP2 (Figure 3C,D). Additionally, miR‐1205 and miR‐382 levels were prominently underexpressed in glioma tissues and cells (Figure 3E–H). Moreover, the levels of miR‐1205 and miR‐382 inversely correlated with circ‐UBAP2 expression in glioma tissues (Figure 3I,J). To elucidate whether circ‐UBAP2 modulated miR‐1205 and miR‐382 expression, we manipulated circ‐UBAP2 expression by si‐circ‐UBAP2 or circ‐UBAP2 overexpressing plasmid in both A172 and U251 cell lines (Figure 3K). As expected, miR‐1205 and miR‐382 levels were significantly increased by circ‐UBAP2 silencing, and they were strongly reduced by the elevated expression of circ‐UBAP2 in the two glioma cell lines (Figure 3L,M). These data together strongly established the notion that circ‐UBAP2 directly targeted miR‐1205 and miR‐382.

### 3.4 miR‐1205 and miR‐382 were two important mediators of circ‐UBAP2 function in modulating cell behaviors in vitro

**FIGURE 4:**
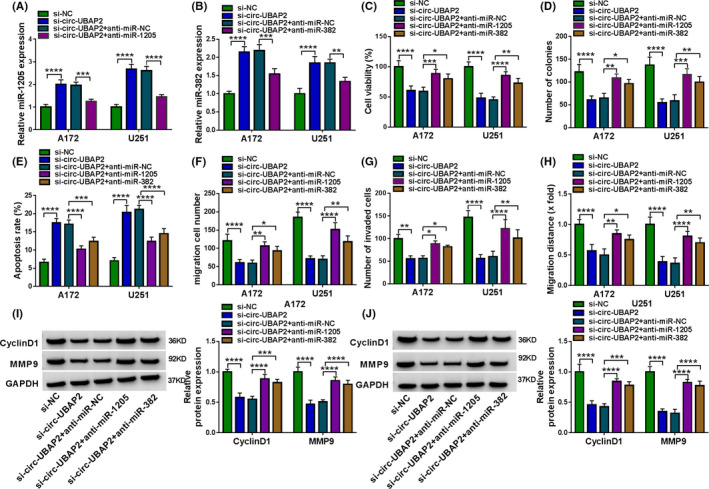
Circ‐UBAP2 modulated glioma cell behaviors via targeting miR‐1205 or miR‐382. A172 and U251 cells were transfected with si‐NC, si‐circ‐UBAP2, si‐circ‐UBAP2+anti‐miR‐NC, si‐circ‐UBAP2+anti‐miR‐1205, or si‐circ‐UBAP2+anti‐miR‐382. (A, B) miR‐1205 and miR‐382 expression were assessed using qRT‐PCR. (C) Cell viability was gauged using CCK‐8 assay. (D) Colony formation was evaluated using colony formation assay. (E) Cell apoptosis was detected using flow cytometry. (F, G) Cell migration and invasion were gauged using transwell assay. (H) Cell migration was monitored using wound‐healing assay. (I, J) The levels of CyclinD1 and MMP9 by western blot. *p < 0.05, **p < 0.01, ***p < 0.001 or ****p < 0.0001

To examine whether circ‐UBAP2 modulated glioma cell behaviors in vitro by the two miRNAs, we used miRNA inhibitors (anti‐miR‐1295 and anti‐miR‐382) to deplete miR‐1205 or miR‐382 in circ‐UBAP2‐silencing A172 and U251 cells. By contrast, circ‐UBAP2 silencing‐mediated augmentation on miR‐1205 and miR‐382 levels was significantly reversed by the corresponding miRNA inhibitor (Figure 4A,B). Moreover, the depletion of miR‐1205 or miR‐382 dramatically abolished circ‐UBAP2 silencing‐mediated viability inhibition (Figure 4C), colony formation repression (Figure 4D and Figure [Link], [Link], [Link], [Link], [Link], [Link], [Link], [Link]A), apoptosis enhancement (Figure 4E and Figure S4B), migration and invasion suppression (Figure 4F–H, and Figure S4C–E). Additionally, the depletion of miR‐1205 or miR‐382 significantly abrogated the inhibition of CyclinD1 and MMP9 levels of circ‐UBAP2 silencing in the two cell lines (Figure 4I,J). Taken together, these results indicated that miR‐1205 and miR‐382 represented two importantly downstream mediators of circ‐UBAP2 function in glioma cell behaviors in vitro.

### 3.5 GPRC5A in glioma cells was directly targeted by miR‐1205 and miR‐382

**FIGURE 5:**
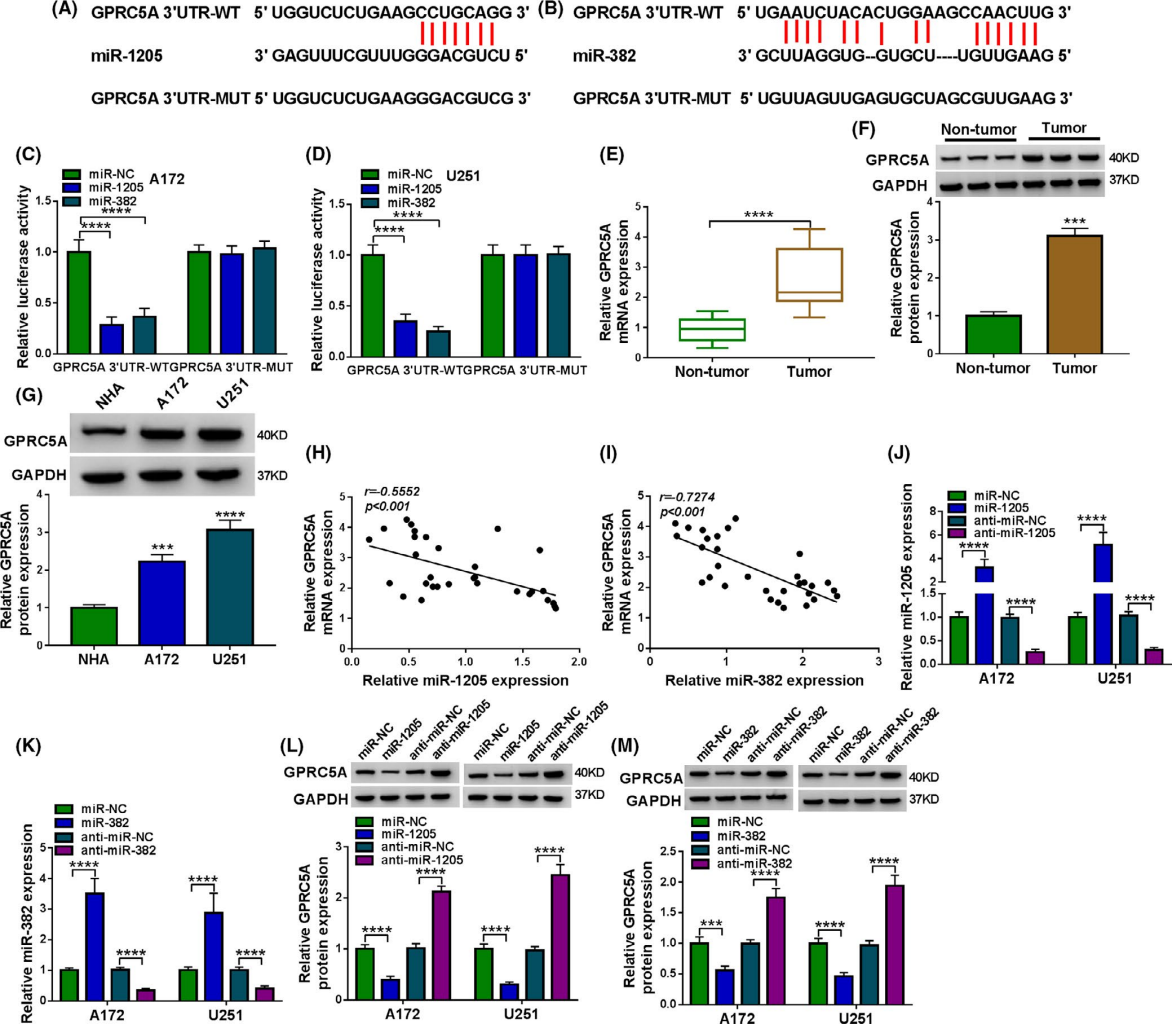
GPRC5A was a direct target of miR‐1205 and miR‐382. (A, B) Nucleotide model of the miR‐1205‐pairing region and the miR‐382‐pairing region with GPRC5A 3'UTR and the mutations of the seed region. (B, C) Dual‐luciferase reporter assays in A172 and U251 cells. (E, F) GPRC5A mRNA and protein levels in tumor tissues and corresponding healthy tissues. (G) GPRC5A protein levels in NHA, A172, and U251 cells. (H, I) Correlation between GPRC5A and miR‐1205 or miR‐382 expression in glioma tissues using the Spearman’s test. (J) miR‐1205 expression in cells transfected with miR‐NC mimic, miR‐1205 mimic, anti‐miR‐NC, or anti‐miR‐1205. (K) miR‐382 expression in cells transfected with miR‐NC mimic, miR‐382 mimic, anti‐miR‐NC, or anti‐miR‐382. (L, M) GPRC5A protein level in cells transfected with miR‐NC mimic, miR‐1205 mimic, miR‐382 mimic, anti‐miR‐NC, anti‐miR‐1205, or anti‐miR‐382. ***p < 0.001 or ****p < 0.0001

Using the starBase software, we selected several genes that were associated with glioma progression. Our data showed that GPRC5A expression was the most significantly downregulated in U251 cells transfected with miR‐1205 or miR‐382 mimic (Figure [Link], [Link], [Link], [Link], [Link], [Link], [Link], [Link]). The predicted data revealed that GPRC5A 3'UTR harbored a putative miR‐1205‐binding sequence and a miR‐382‐binding sequence (Figure 5A,B). The transfection of miR‐1205 or miR‐382 mimic significantly decreased the luciferase activity of GPRC5A 3'UTR wild‐type reporter (GPRC5A 3'UTR‐WT, Figure 5C,D). When the target sequence was mutated, no reduction was observed in luciferase with miR‐1205 mimic or miR‐382 mimic (Figure 5C,D). Additionally, by contrast, GPRC5A was prominently overexpressed in glioma tissues and cell lines (Figure 5E–G). Interestingly, GPRC5A expression inversely correlated with miR‐1205 and miR‐382 levels in tumor tissues (Figure 5H,I). We then assessed whether miR‐1205 and miR‐382 could regulate GPRC5A expression. The transfection efficiencies of miR‐1205 mimic, anti‐miR‐1205, miR‐382 mimic, and anti‐miR‐382 were confirmed by qRT‐PCR in the two glioma cell lines (Figure 5J,K). As expected, GPRC5A protein expression was significantly reduced by the overexpression of miR‐1205 or miR‐382, while it was dramatically augmented by miR‐1205 or miR‐382 depletion (Figure 5L,M). Together, these results demonstrated that GPRC5A was a direct target of miR‐1205 and miR‐382.

### 3.6 GPRC5A was a functional target of miR‐1205 and miR‐382 in regulating cell behaviors in vitro

**FIGURE 6:**
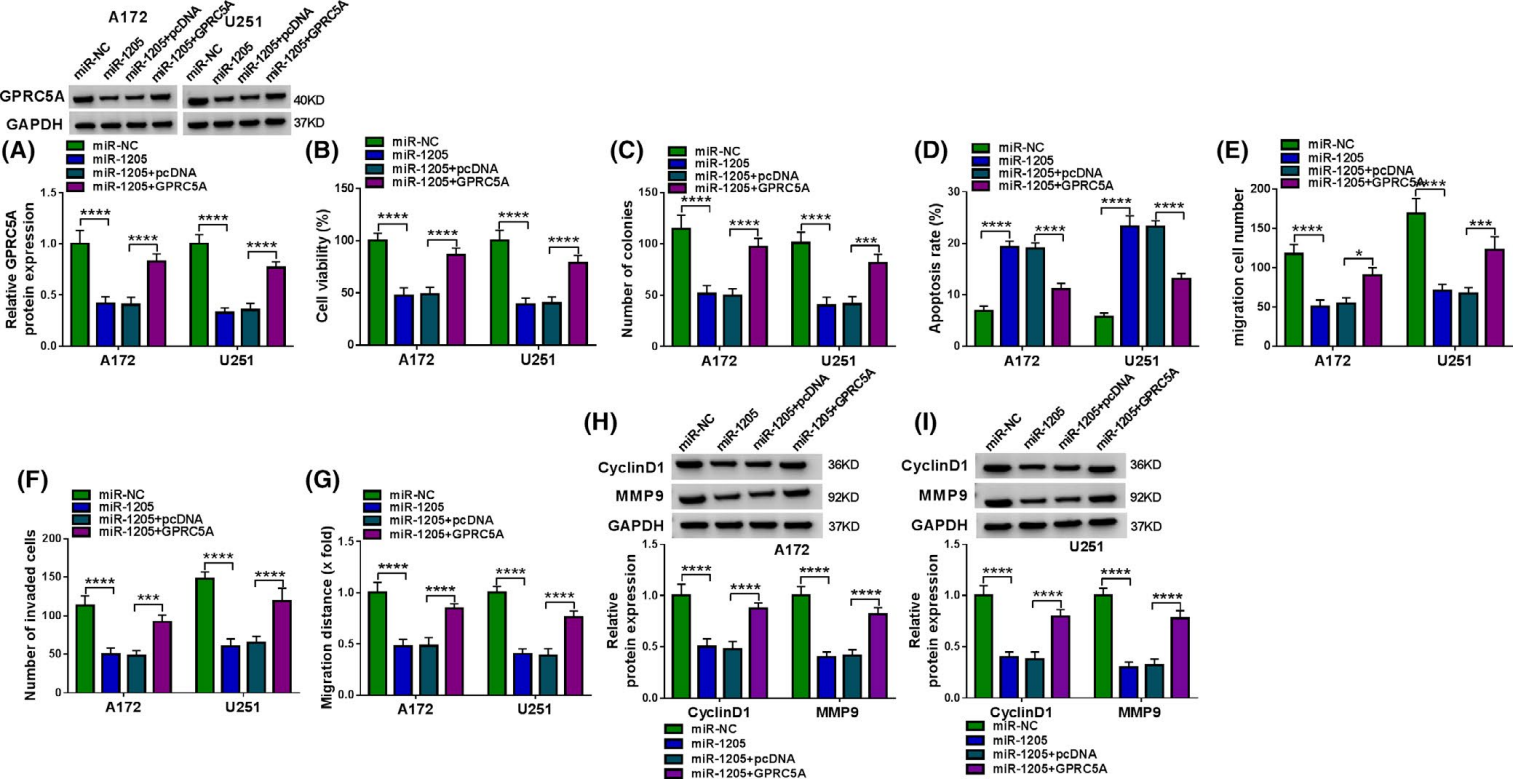
miR‐1205 overexpression regulated cell behaviors by downregulating GPRC5A. A172 and U251 cells were transfected with miR‐NC mimic, miR‐1205 mimic, miR‐1205 mimic+pcDNA, or miR‐1205 mimic+GPRC5A (GPRC5A overexpressing plasmid). (A) GPRC5A protein expression was assessed using western blot. (B) Cell viability was gauged using CCK‐8 assay. (C) Colony formation was evaluated using colony formation assay. (D) Cell apoptosis was detected using flow cytometry. (E, F) Cell migration and invasion were gauged using transwell assay. (G) Cell migration was monitored using wound‐healing assay. (H, I) The levels of CyclinD1 and MMP9 by western blot. *p < 0.05, ***p < 0.001, or ****p < 0.0001

In order to elucidate the link between GPRC5A and miR‐1205 or miR‐382 in glioma progression, we overexpressed GPRC5A in cells transfected with miR‐1205 or miR‐382 mimic. In addition to the reduced impact on GPRC5A expression (Figure 6A), the increased expression of miR‐1205 strikingly inhibited cell viability (Figure 6B), colony formation (Figure 6C and Figure [Link], [Link], [Link], [Link], [Link], [Link], [Link], [Link]A), and enhanced apoptosis (Figure 6D and Figure S6B), as well as suppressed migration and invasion (Figure 6E–G, and Figure S6C–E). Additionally, the overexpression of miR‐1205 prominently reduced the expression of CyclinD1 and MMP9 in both A172 and U251 cell lines (Figure 6H,I). “Rescue” experiments wherein GPRC5A was upregulated using an overexpressing plasmid (Figure 6A) dramatically abolished the regulation of miR‐1205 overexpression on cell functional behaviors (Figure 6B–I, and Figure S6A–E).

**FIGURE 7:**
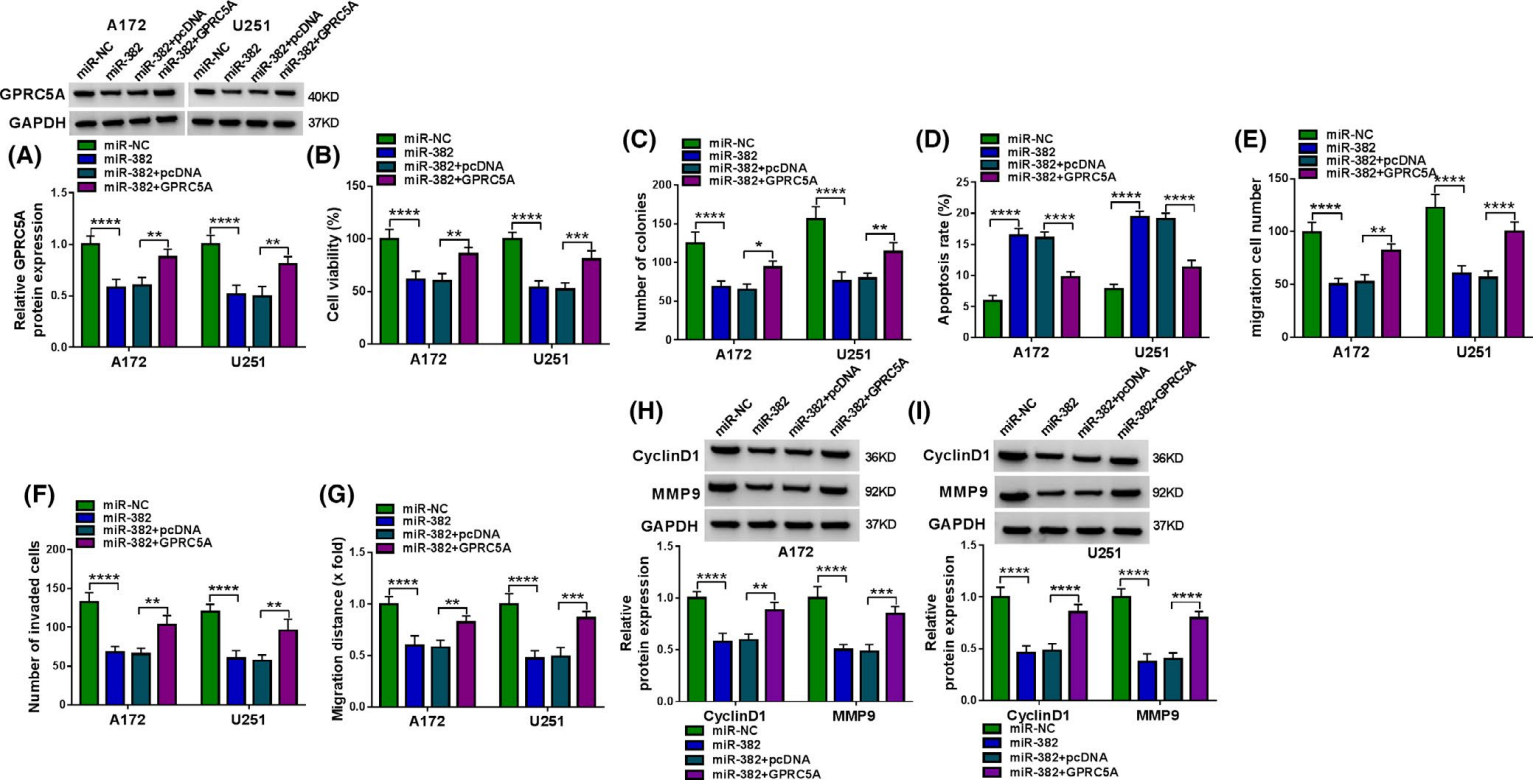
The regulation of miR‐382 on cell proliferation, migration, invasion, and apoptosis was mediated by GPRC5A. A172 and U251 cells were transfected with miR‐NC mimic, miR‐382 mimic, miR‐382 mimic+pcDNA, or miR‐382 mimic+GPRC5A (GPRC5A overexpressing plasmid). (A) GPRC5A protein expression was assessed using western blot. (B) Cell viability was gauged using CCK‐8 assay. (C) Colony formation was evaluated using colony formation assay. (D) Cell apoptosis was detected using flow cytometry. (E, F) Cell migration and invasion were gauged using a transwell assay. (G) Cell migration was monitored using a wound‐healing assay. (H, I) The levels of CyclinD1 and MMP9 by western blot. *p < 0.05, **p < 0.01, ***p < 0.001, or ****p < 0.0001

Similarly, the transfection of GPRC5A overexpressing plasmid reversed miR‐382 overexpression‐mediated GPRC5A downregulation in the two glioma cell lines (Figure 7A). Importantly, the elevated level of GPRC5A remarkably abrogated miR‐382 overexpression‐mediated viability repression (Figure 7B), colony formation inhibition (Figure 7C and Figure [Link], [Link], [Link], [Link], [Link], [Link], [Link], [Link]A), apoptosis promotion (Figure 7D and Figure S7B), migration and invasion suppression (Figure 7E–G, and Figure S7C–E), as well as CyclinD1 and MMP9 expression inhibition (Figure 7H,I). Taken together, these results suggested that miR‐1205 and miR‐382 regulated the function behaviors of glioma cells in vitro by targeting GPRC5A.

### 3.7 Circ‐UBAP2 controlled GPRC5A expression by sponging miR‐1205 or miR‐382

**FIGURE 8:**
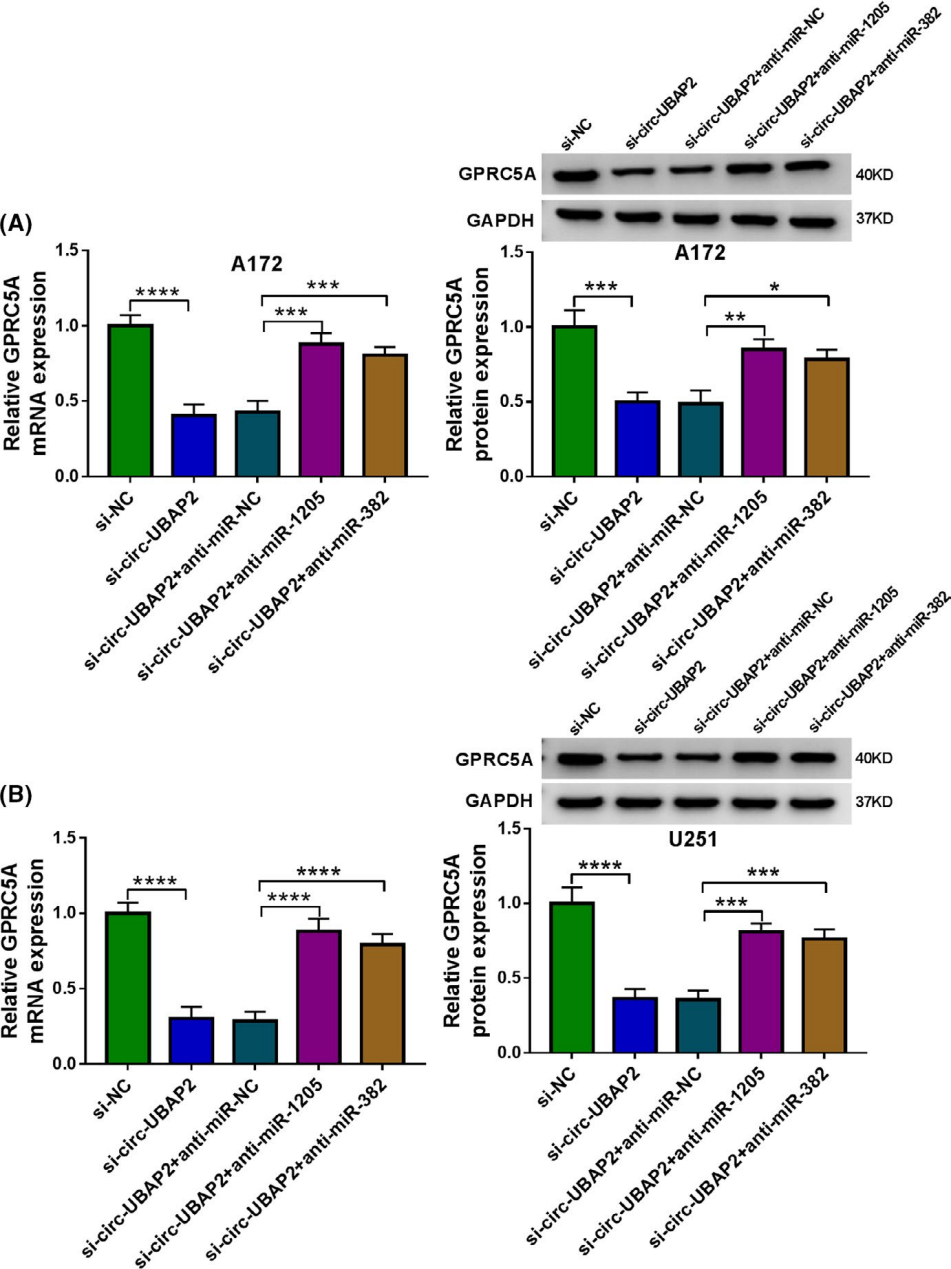
Circ‐UBAP2 functioned as sponges of miR‐1205 and miR‐382 to control GPRC5A expression. A172 (A) and U251 (B) cells were transfected with si‐NC, si‐circ‐UBAP2, si‐circ‐UBAP2+anti‐miR‐NC, si‐circ‐UBAP2+anti‐miR‐1205 or si‐circ‐UBAP2+anti‐miR‐382, followed by the measurement of GPRC5A mRNA and protein levels by qRT‐PCR and western blot. *p < 0.05, **p < 0.01, ***p < 0.001, or ****p < 0.0001

We then determined whether circ‐UBAP2 could influence GPRC5A expression. By contrast, UBAP2 silencing resulted in decreased expression of GPRC5A in both A172 and U251 cell lines (Figure 8A,B). Furthermore, this effect was prominently reversed by anti‐miR‐1205 or anti‐miR‐382 transfection (Figure 8A,B). These data together strongly pointed to the role of circ‐UBAP2 as a regulator of GPRC5A expression through miR‐1205 and miR‐382.

### 3.8 Silencing of circ‐UBAP2 declined tumor growth in vivo

**FIGURE 9:**
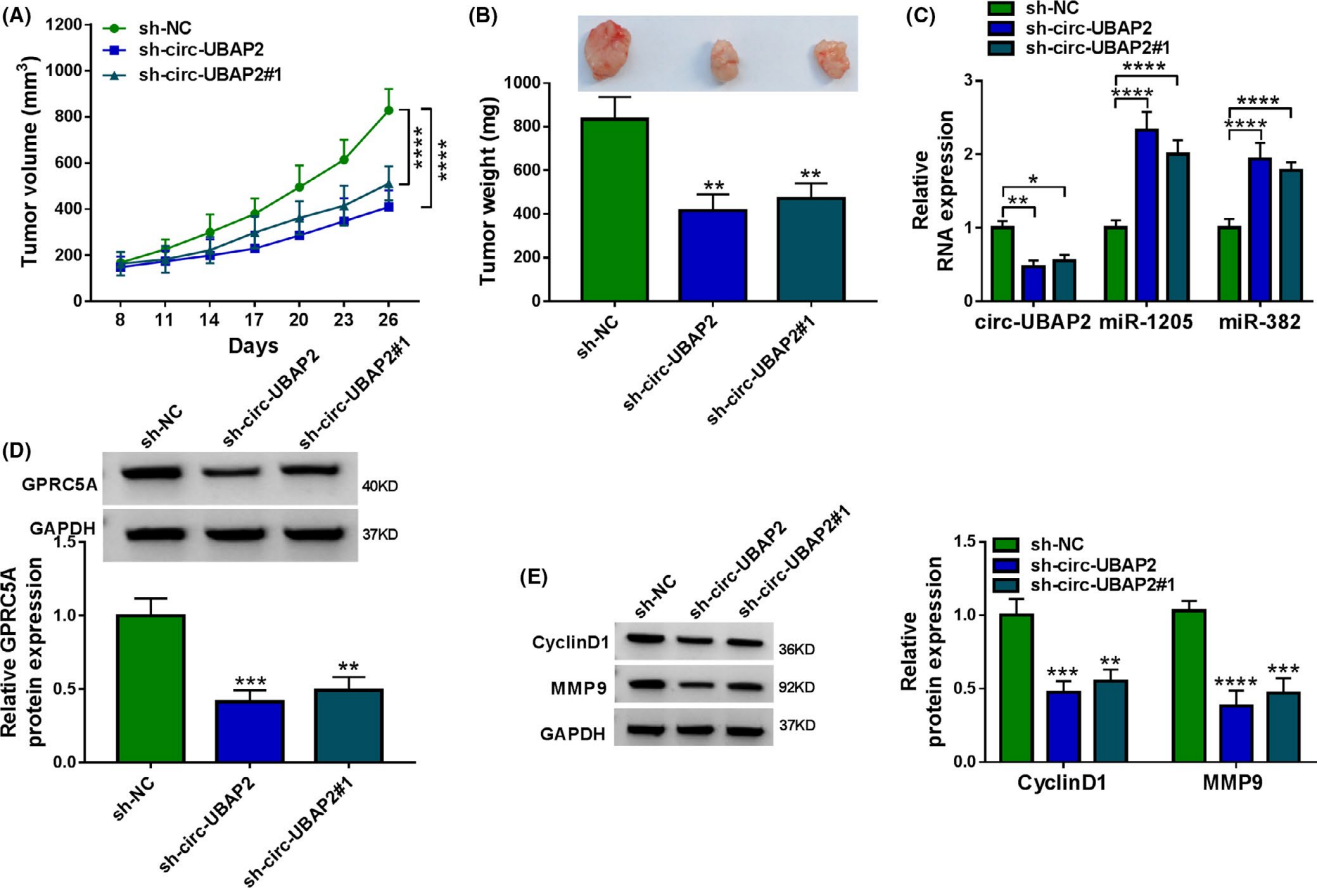
Circ‐UBAP2 silencing repressed tumor growth in vivo. sh‐circ‐UBAP2‐, sh‐circ‐UBAP2#1‐transduced, or sh‐NC‐infected U251 cells were implanted into the nude mice (*n* = 6). (A) Growth curves of the xenograft tissues. (B) Representative pictures and average weight of the tumors tissues. The levels of circ‐UBAP2, miR‐1205, and miR‐382 were measured using qRT‐PCR (C), and GPRC5A protein expression (D) and CyclinD1 and MMP9 levels (E) by western blot in the excised tumor tissues. *p < 0.05, **p < 0.01, ***p < 0.001, or ****p < 0.0001

An important question was whether circ‐UBAP2 could modulate tumor growth in vivo. To elucidate this, we implanted sh‐circ‐UBAP2‐, sh‐circ‐UBAP2#1‐transduced, or sh‐NC‐infected U251 cells into the nude mice to generate the xenograft model. In contrast, the transduction of sh‐circ‐UBAP2 or sh‐circ‐UBAP2#1 caused a remarkable repression in tumor growth (Figure 9A,B). Furthermore, the levels of circ‐UBAP2 and GPRC5A were remarkably reduced and the expression levels of miR‐1205 and miR‐382 were strongly augmented in the xenograft tissues derived from sh‐circ‐UBAP2‐transduced U251 cells (Figure 9C,D). Moreover, the silencing of circ‐UBAP2 led to a clear reduction in the levels of CyclinD1 and MMP9 in the xenograft tumors (Figure 9E). Additionally, the restored expression of circ‐UBAP2 in the xenograft tumors significantly abrogated the impact of sh‐circ‐UBAP2 on tumor growth and miR‐1205 and miR‐382 expression (Figure S2H–J). All these results suggested that circ‐UBAP2 silencing diminished tumor growth in vivo.

## 4 DISCUSSION

Emerging evidence is pointing toward circRNAs as tumor suppressors and oncogenic drivers in glioma.^5^ Furthermore, the precise parts of these circRNAs in glioma development largely remain to be elucidated. Considering the oncogenic regulation of circ‐UBAP2 in glioma,^8^ we here sought to identify the molecular basis governing it.

In this report, our data supported the significant overexpression of circ‐UBAP2 in glioma.^8^ Consistent with a previous study,^8^ we demonstrated that circ‐UBAP2 silencing performed an antitumor activity in glioma in vitro and in vivo. As has been reported for other circRNAs,^19^, ^20^ circ‐UBAP2 was resistant to RNase R because circRNAs have no free 3' or 5' end. Furthermore, circ‐UBAP2 was mainly present in the cytoplasm, offering the possibility for its interaction with miRNAs. Our results also showed that the overexpression of circ‐UBAP2 did not affect cell colony formation, migration, invasion, and apoptosis (Figure [Link], [Link], [Link], [Link], [Link], [Link], [Link], [Link]), which was different to glioma cells. This might be due to the dysregulation of circ‐UBAP2 on cancer cells.

Using CircInteractome prediction tool, we confirmed that circ‐UBAP2 directly targeted miR‐1205 and miR‐382, which had been highlighted as tumor inhibitors in glioma.^11^, ^12^, ^13^, ^14^, ^21^ Previous work uncovered the critical involvement of miR‐1205 in many human tumors, including laryngeal squamous cell carcinoma and prostate cancer.^22^, ^23^ miR‐382 was illuminated as an essential player in pancreatic cancer and breast cancer.^24^, ^25^ Here, we first demonstrated that miR‐1205 and miR‐382 were two molecular mediators of circ‐UBAP2 in modulating glioma cell progression. Similarly, several other circRNAs, such as hsa_circ_0034642 and circ‐POSTN, contributed to glioma carcinogenesis by targeting miR‐1205.^12^, ^21^ He et al. identified that circ‐DICER1 operated as a miR‐382 sponge to control glioma angiogenesis.^14^

Abnormal expression of GPRC5A has been unraveled in various types of tumors, such as prostate cancer, gastric cancer and colorectal cancer.^26^, ^27^, ^28^ A previous study demonstrated the oncogenic role of GPRC5A in glioma.^29^ Interestingly, we first highlighted that GPRC5A was an importantly functional target of miR‐1205 and miR‐382 in suppressing glioma cell progression. Similarly, Wang et al. demonstrated that miR‐342 suppressed the progression of glioma through inhibiting GPRC5A.^29^ Furthermore, we were first to uncover that circ‐UBAP2 mediated GPRC5A expression through miR‐1205 or miR‐382. Such analysis was hampered at present by the lack of direct evidence about the novel mechanisms in glioma tumor growth in vivo.

In summary, our current work demonstrated that circ‐UBAP2 silencing impeded glioma malignant progression by downregulating GPRC5A by targeting miR‐1205 and miR‐382. Here, we identified two novel mechanisms, the circ‐UBAP2/miR‐1205/GPRC5A and circ‐UBAP2/miR‐382/GPRC5A axes, in the development of glioma, highlighting circ‐UBAP2 inhibitor as a potential therapeutic strategy for glioma.

## CONFLICT OF INTEREST

The authors declare that they have no financial conflicts of interest.

## AUTHOR CONTRIBUTIONS

Jianxin Wang conceived and designed the experiments, wrote the paper; Tianxiao Li performed the experiments; Bin Wang contributed reagents/materials/analysis tools. All authors read and approved the final manuscript.

## Supporting information


**Figure S1.**

**Figure S2.**

**Figure S3.**

**Figure S4.**

**Figure S5**.

**Figure S6.**

**Figure S7**.

**Figure S8.**
