# Supplementary figures and images for: Circ‐UBAP2 functions as sponges of miR‐1205 and miR‐382 to promote glioma progression by modulating STC1 expression
Source: Cancer Med. 2021 Feb 5;10(5):1815–28. doi: 10.1002/cam4.3759 (PMC7940226; doi:10.1002/cam4.3759)

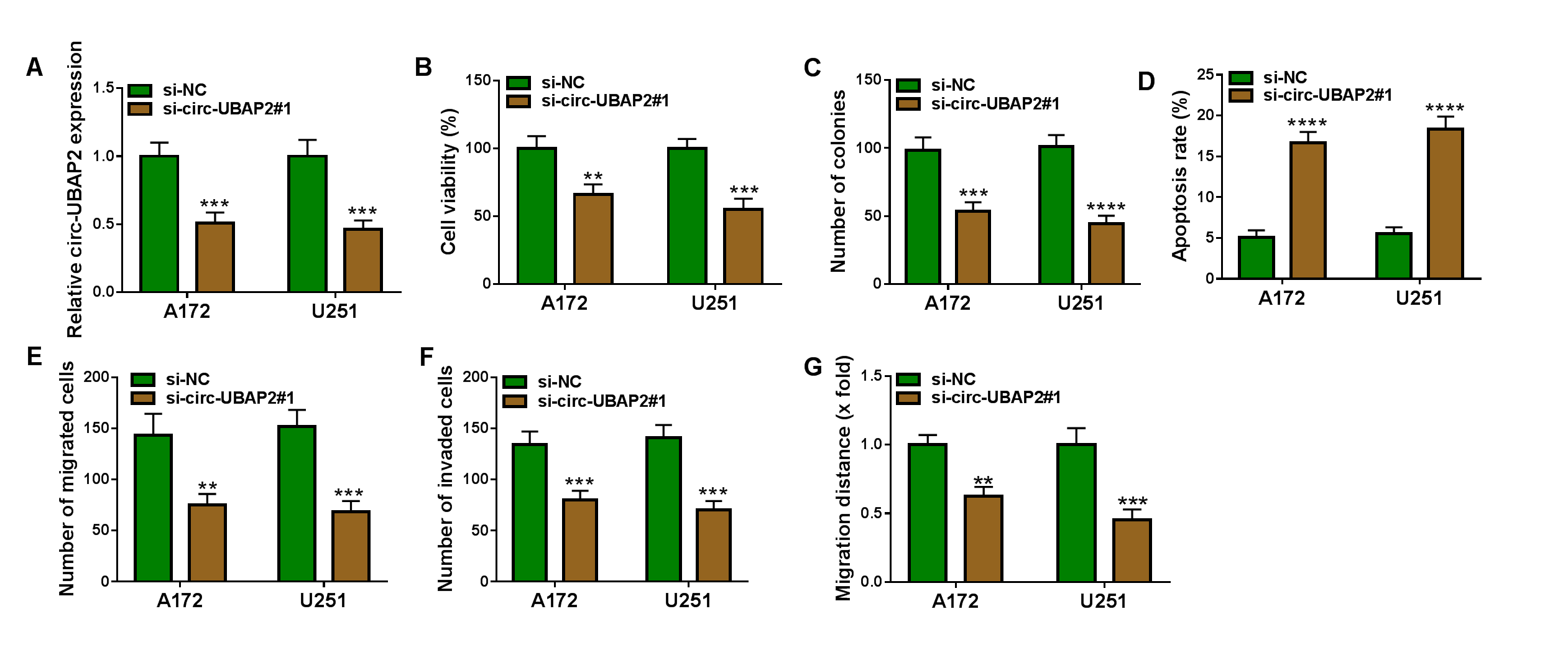

Supplement: Supplementary file 1 — Figure S1. [file CAM4-10-1815-s002.tif]

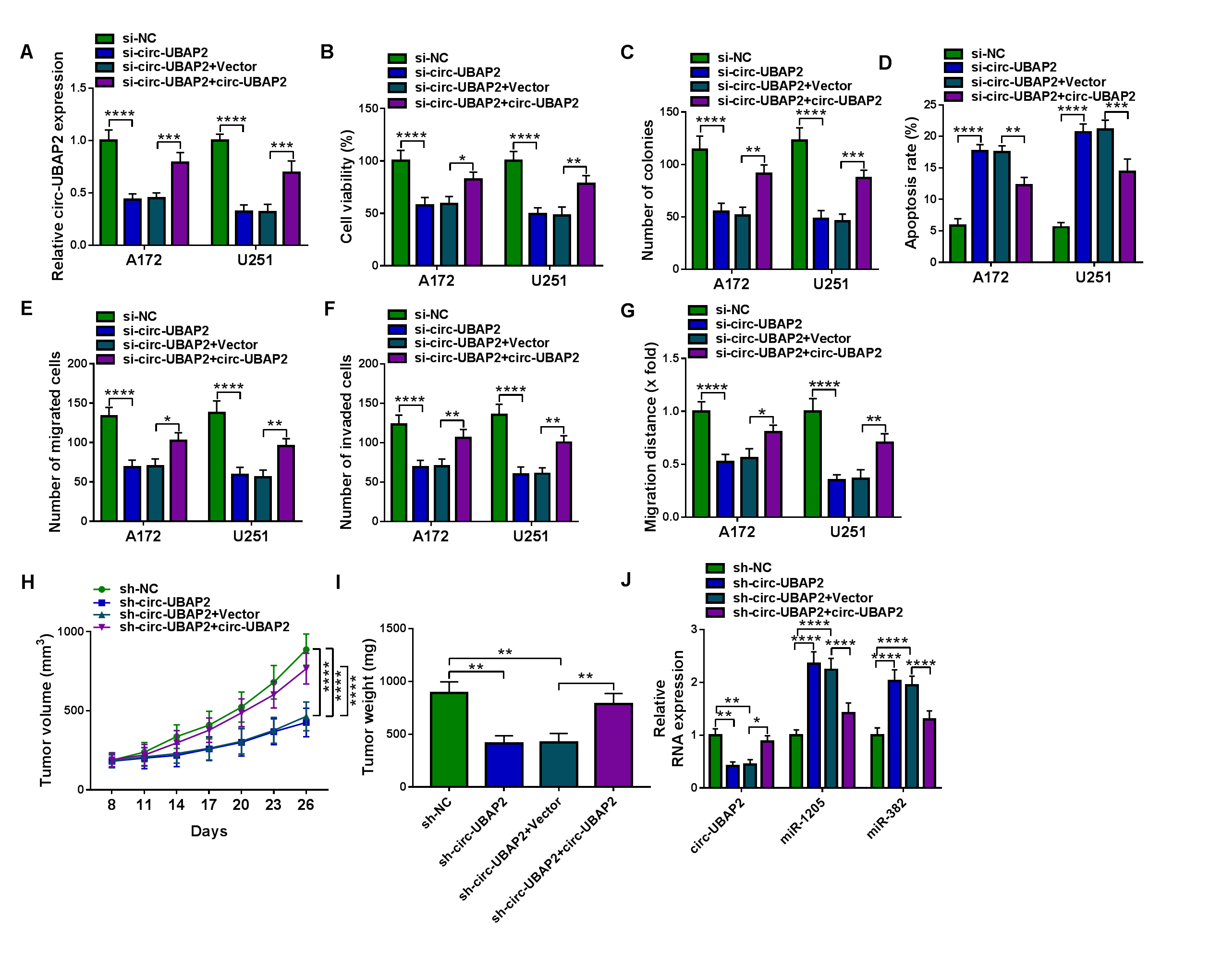

Supplement: Supplementary file 2 — Figure S2. [file CAM4-10-1815-s008.tif]

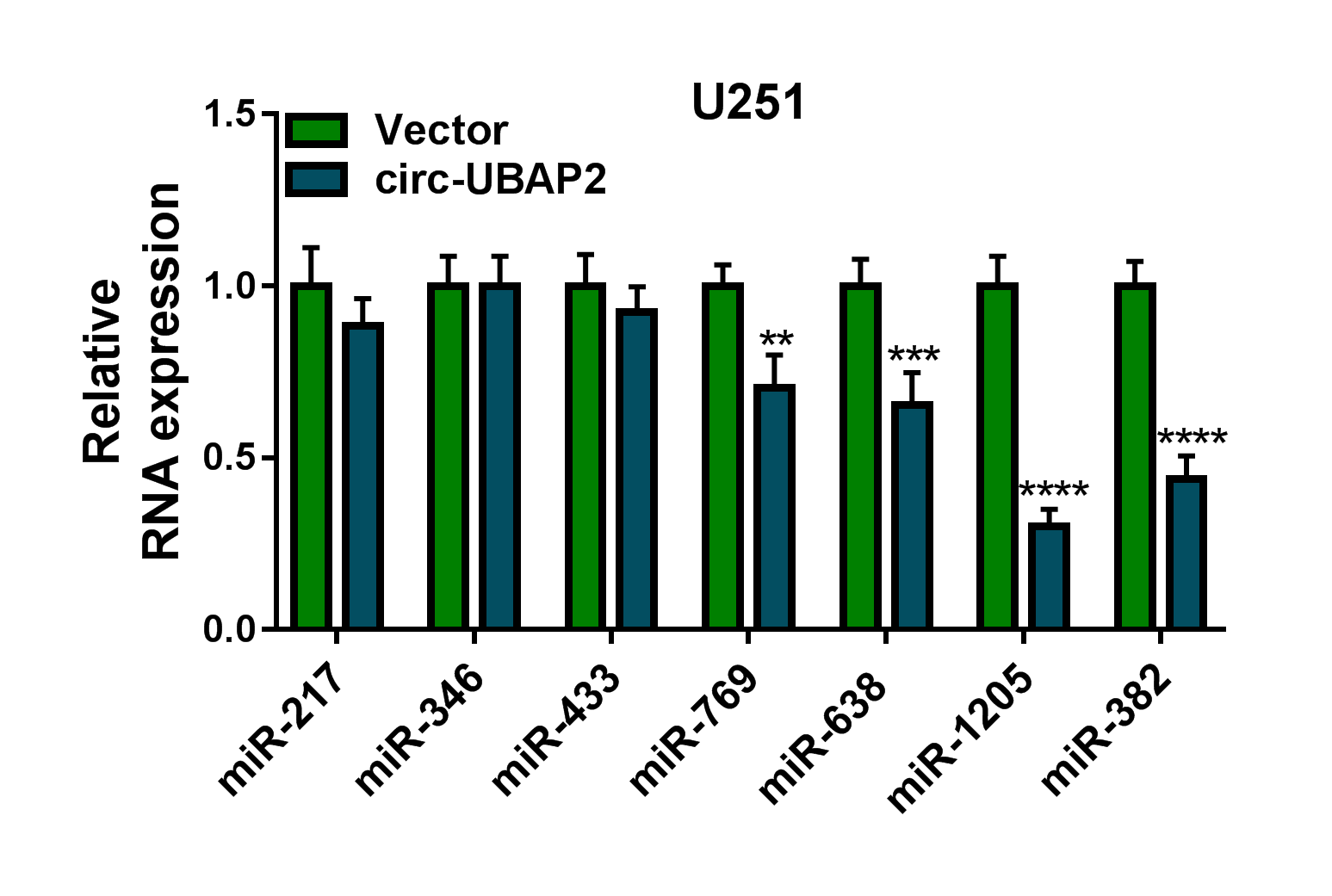

Supplement: Supplementary file 3 — Figure S3. [file CAM4-10-1815-s001.tif]

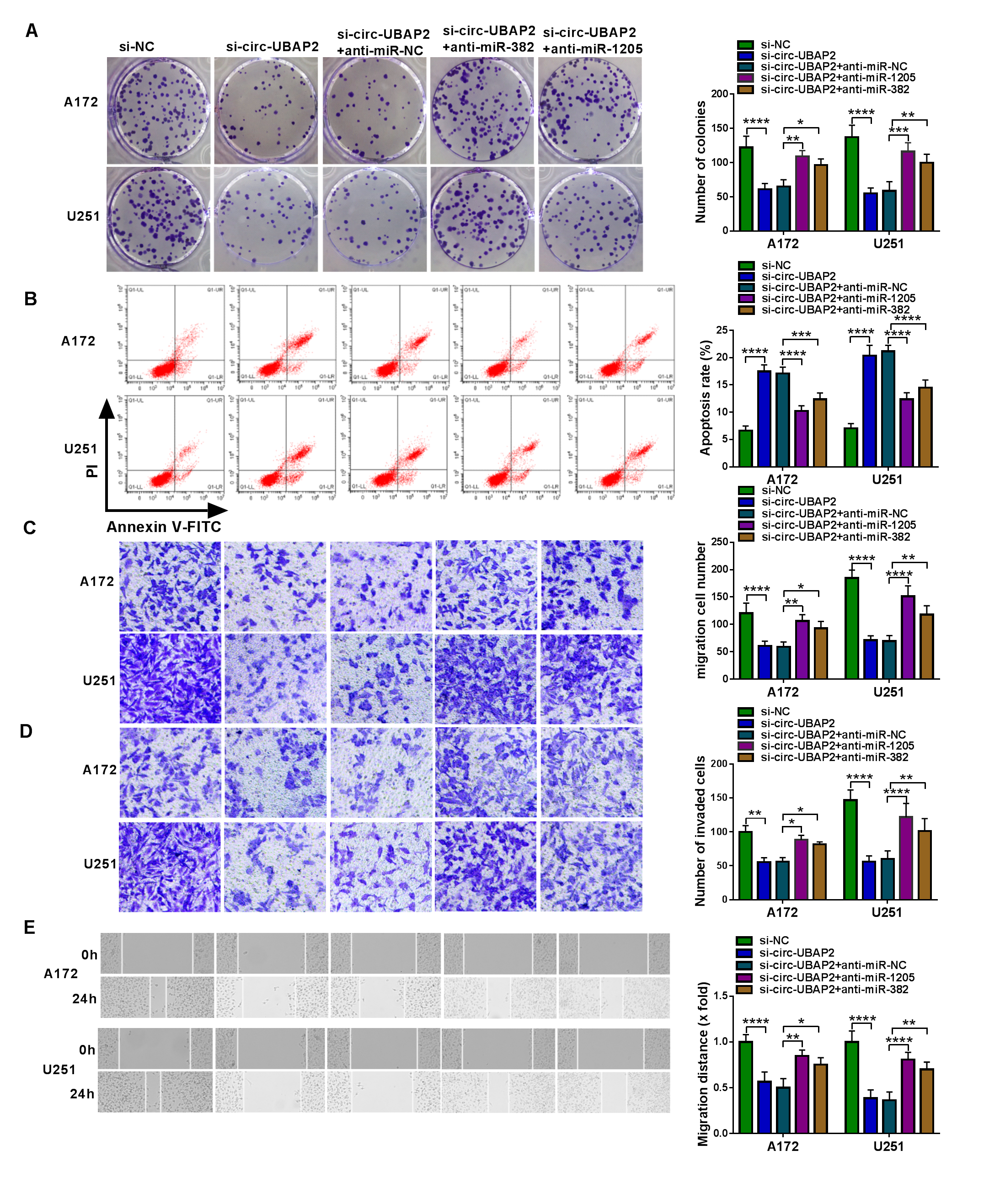

Supplement: Supplementary file 4 — Figure S4. [file CAM4-10-1815-s006.tif]

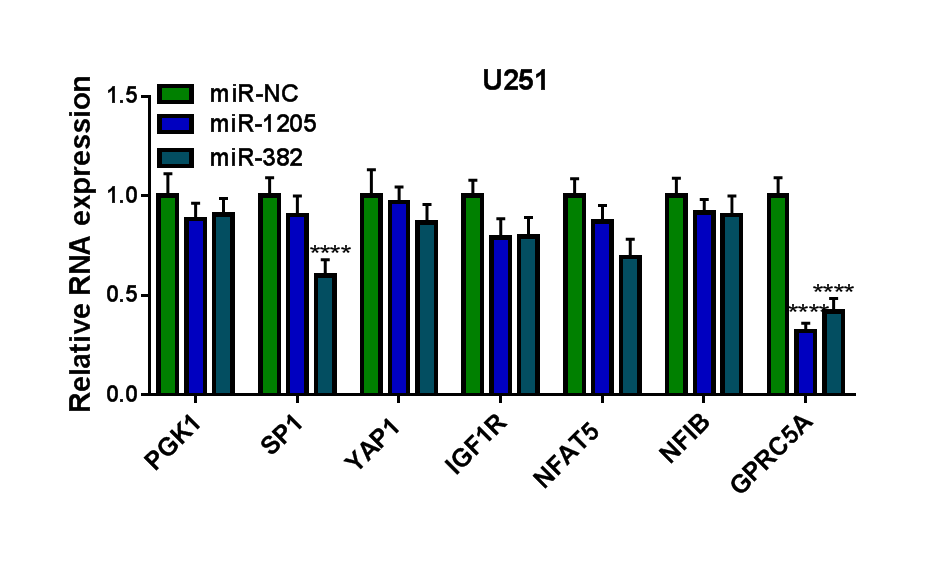

Supplement: Supplementary file 5 — Figure S5. [file CAM4-10-1815-s005.tif]

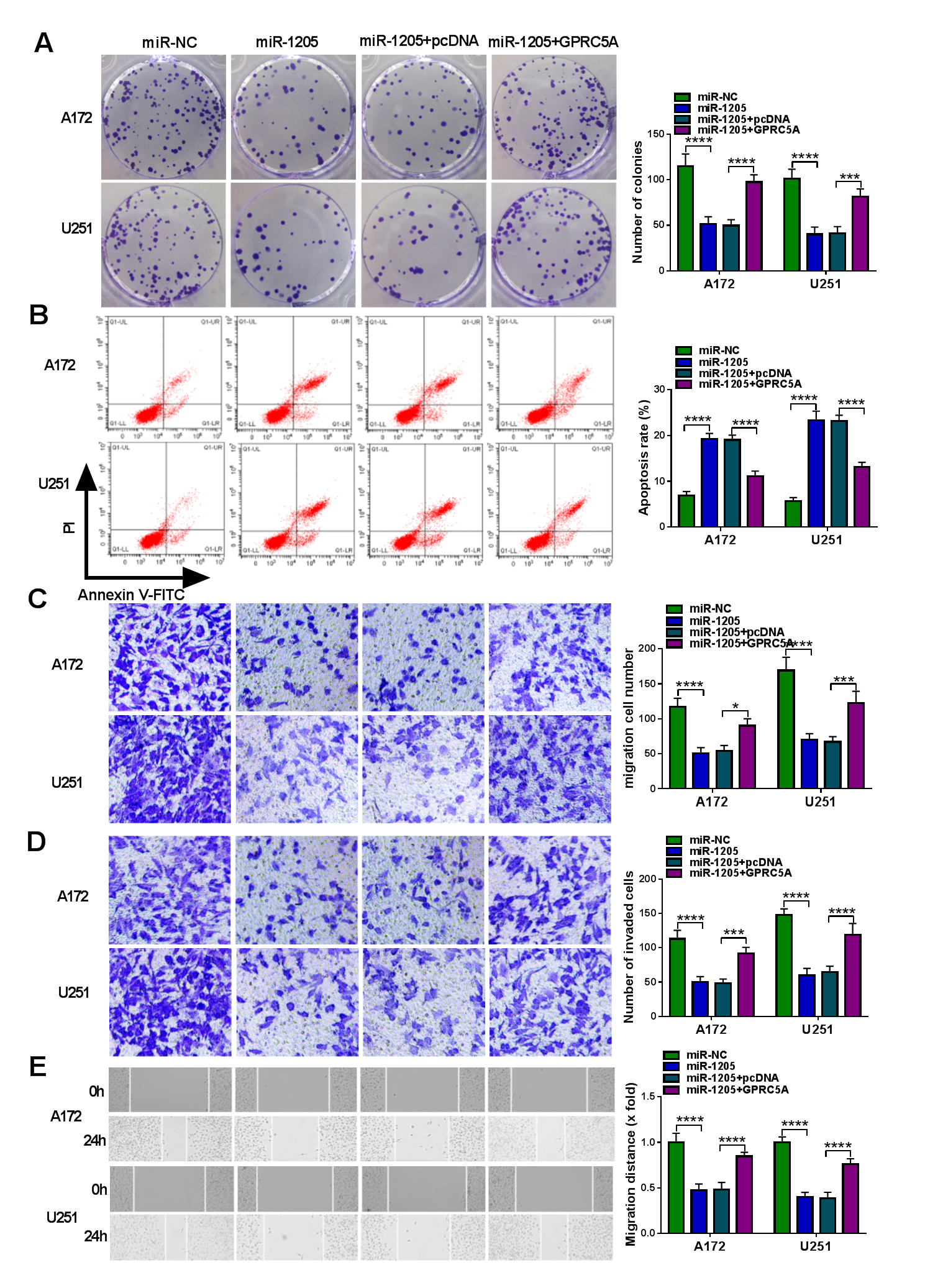

Supplement: Supplementary file 6 — Figure S6. [file CAM4-10-1815-s007.tif]

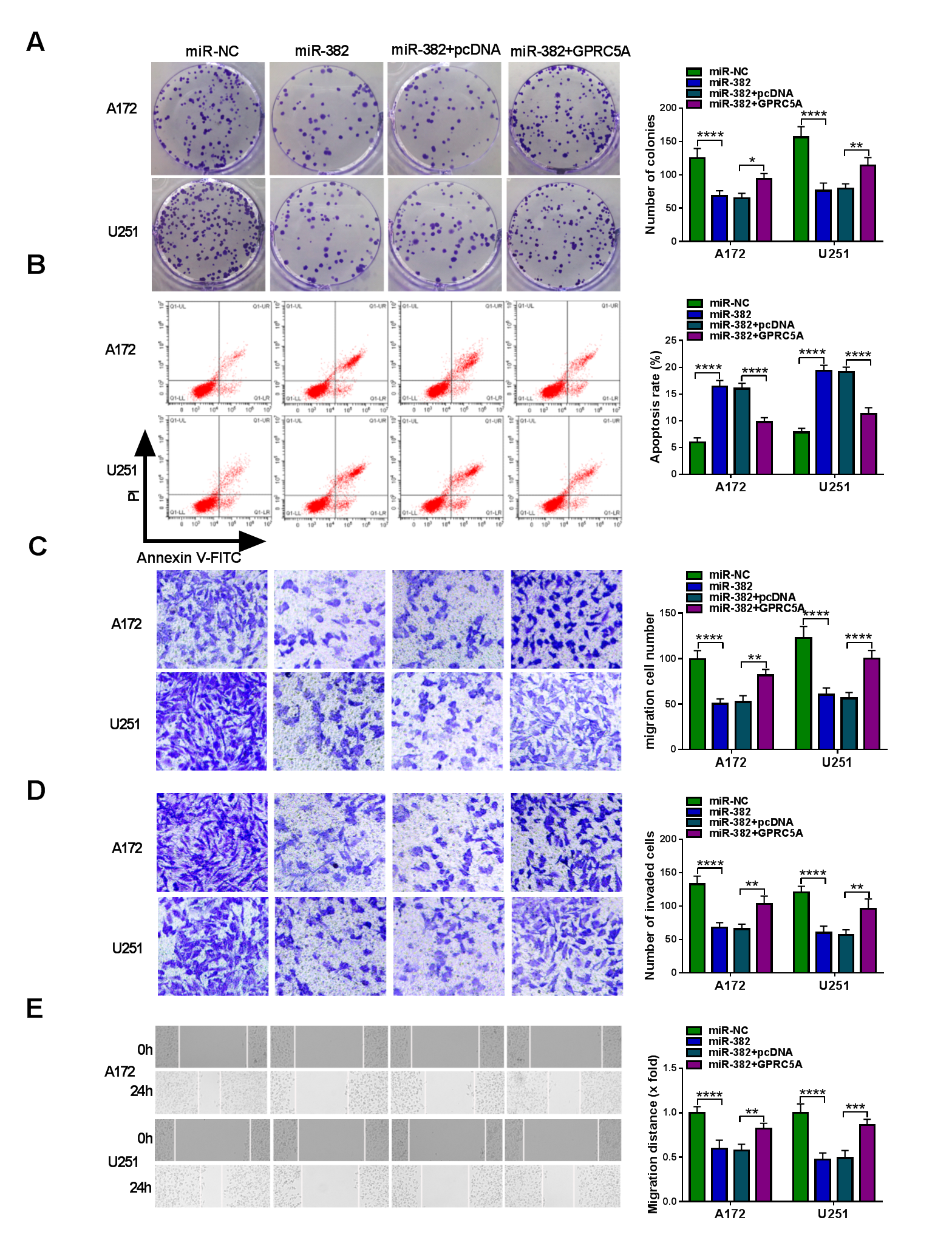

Supplement: Supplementary file 7 — Figure S7. [file CAM4-10-1815-s004.tif]

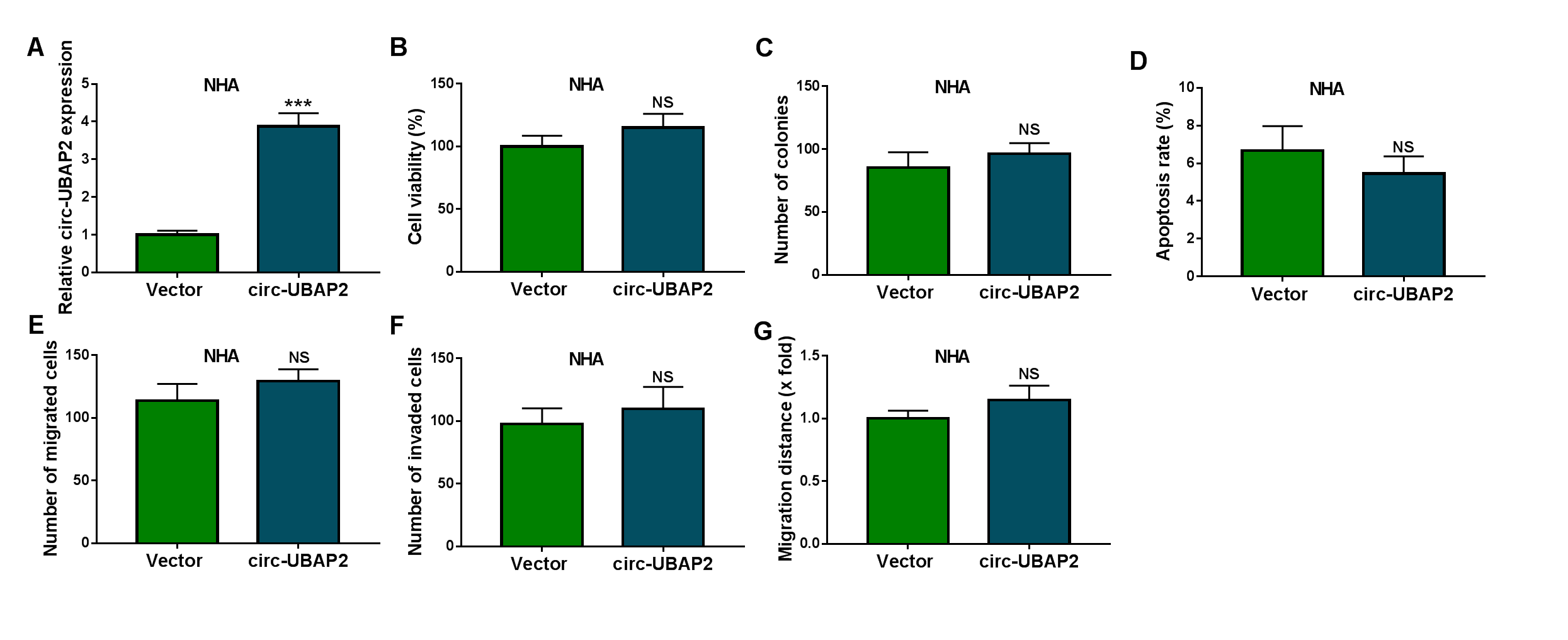

Supplement: Supplementary file 8 — Figure S8. [file CAM4-10-1815-s003.tif]
